# Protein kinase D1 phosphorylation of KAT7 enhances its protein stability and promotes replication licensing and cell proliferation

**DOI:** 10.1038/s41420-020-00323-w

**Published:** 2020-09-18

**Authors:** Yao Liang, Yuanyuan Su, Chenzhong Xu, Na Zhang, Doudou Liu, Guodong Li, Tanjun Tong, Jun Chen

**Affiliations:** Peking University Research Center on Aging, Beijing Key Laboratory of Protein Posttranslational Modifications and Cell Function, Department of Biochemistry and Molecular Biology, Department of Integration of Chinese and Western Medicine, School of Basic Medical Science, Peking University, Beijing, 100191 China

**Keywords:** Kinases, Chemical modification

## Abstract

The histone acetyltransferase (HAT) KAT7/HBO1/MYST2 plays a crucial role in the pre-replication complex (pre-RC) formation, DNA replication and cell proliferation via acetylation of histone H4 and H3. In a search for protein kinase D1 (PKD1)-interacting proteins, we have identified KAT7 as a potential PKD1 substrate. We show that PKD1 directly interacts and phosphorylates KAT7 at Thr97 and Thr331 in vitro and in vivo. PKD1-mediated phosphorylation of KAT7 enhances its expression levels and stability by reducing its ubiquitination-mediated degradation. Significantly, the phospho-defective mutant KAT7-Thr97/331A attenuates histone H4 acetylation levels, MCM2/6 loading on the chromatin, DNA replication and cell proliferation. Similarly, PKD1 knockdown decreases, whereas the constitutive active mutant PKD1-CA increases histone H4 acetylation levels and MCM2/6 loading on the chromatin. Overall, these results suggest that PKD1-mediated phosphorylation of KAT7 may be required for pre-RC formation and DNA replication.

## Introduction

The Protein kinase D (PKD) is a serine/threonine-specific protein kinase family that belongs to the calcium/calmodulin-dependent kinase (CAMK) family and composes of PKD1, PKD2, and PKD3 three isoforms^1–3^. PKD is activated mostly by cell growth promoting substances, such as phorbol ester, platelet derived growth factor and G protein-couple receptor (GPCR) ligands^2^. PKD1 activation is marked by phosphorylation of two conserved serine residues Ser738 and Ser742 within the activation loop and concomitant auto-phosphorylation of ser916^2^. PKD1 has been implicated in diverse cellular processes such as vesicle fission and protein secretion from trans-Golgi network to cell surface^4,5^, cell proliferation^6–8^, cell adhesion and motility^9,10^, oxidative stress signaling^11,12^, immune responses^13^ and cancer^14–16^. As a protein kinase, phosphorylation of its substrates is the major molecular mechanism for PKD1 mediating various biological functions.

KAT7, a member of the MYST family of histone acetyltransferases (HAT), is originally identified through its interaction with the origin recognition complex 1 (ORC1)^17^, also known as MYST2 or HBO1 (histone acetyltransferase binding to ORC1). As a histone acetylation enzyme, KAT7 associates with distinct protein complexes to modulate DNA replication and cell cycle progression via epigenetic mechanisms^18–20^. A key step in DNA replication is the formation of a pre-replication complex (pre-RC) involving the sequential assembly of the ORC1, Cdc6/Cdcl8, Cdt1, and the mini-chromosome maintenance (MCM2-7) complex^21^. KAT7 initiates DNA replication by acetylating both histone H3 and H4. When KAT7 complexes with ING4/5 and JADE1/2/3, it preferentially acetylates histone H4 at lysine 5, 8 and 12 around replication origins to facilitate pre-RC formation at G1 phase^20,22^. However, when KAT7 complexes with ING4/5 and BRPF1/2/3, it preferentially acetylates histone H3 at K14 instead of H4 to regulate replication origin activation at S phase^23–25^. KAT7-JADE-ING complexes also directly bind and stimulate p53-regulated genes to control cell proliferation^26^.

Several lines of evidence have suggested that KAT7 is subjected to the posttranslational modifications, particularly phosphorylation modification, in response to the environment or cell signaling cues. For example, Polo-like kinase (PLK1)-mediated phosphorylation of KAT7 at Ser57 is required for pre-RC formation and DNA replication licensing^27^. UV damage induced phosphorylation of KAT7 at Ser50/53 by ATM/ATR leads to KAT7 degradation and suppresses cell proliferation and facilitates nucleotide excision repair^28,29^. The cyclin E/CDK2 complex phosphorylates KAT7 at Thr88 which promotes the enrichment of cancer stem-like cells in breast cancer^30^. Lipopolysaccharide (LPS)-induced phosphorylation of KAT7 by mitogen-activated protein kinase 1 (MEK1) triggers KAT7 degradation by ubiquitin E3 ligase FBXW15 and prevents cell proliferation^31^. However, the regulative mechanisms and functions of KAT7 phosphorylation is still limited.

In this study, we demonstrate that PKD1 associates with KAT7 and phosphorylates KAT7 at Thr97 and Thr331. PKD1-mediated phosphorylation of KAT7 enhances its protein expression levels and stability by inhibiting KAT7 ubiquitination. Moreover, KAT7 phosphorylation by PKD1 promotes DNA replication and cell proliferation.

## Materials and methods

### Cell culture

HEK293T and HeLa cells were cultured in DMEM containing 10% fetal bovine serum (FBS) at 37 °C. H1299 cells were cultured in RPMI 1640 medium supplemented with 10% FBS at 37 °C.

### Antibodies

The following primary antibodies were used for immunoblotting analysis: anti-KAT7 (ab190908, Abcam), anti-PKD1 (90039S, Cell Signaling Technology-CST), anti-HA (CST, 3724S), anti-Flag (Sigma, F7425), anti-pMOTIF PKD1 (4381, CST), anti-phosphoserine (ab9332, Abcam), anti-phosphothreonine (CST, 9381S), anti-Tubulin (BS1482M, BioWorld), anti-phospho-PKD1(ser744/748) (CST, 2054), anti-GAPDH (Bioworld, AP0063), anti-Myc, anti-GST(Santa Cruz, sc-965), anti-mcm2 (ab133325, Abcam), anti-mcm6 (ab201683, Abcam), anti-H4 (13919S, CST), anti-H4acK5, K8, K12, K16 (ab177790, Abcam), anti-H3.

### Plasmids and siRNAs transfection

Full-length KAT7 and KAT7 mutant plasmids were transiently transfected into cells with PEI Reagent following the manufacturer’s instruction. Two independent siRNA sequences against PKD1 were: siRNA#1: 5′-GUCGAGAGAAGAGGUCAAATT-3′, and siRNA#2: 5′-CAAUCCUCAUUGUUUCGAAAT-3′. The siRNA sequence against KAT7 mRNA 3’-UTR was: 5’-GCUGCAUAUUAACUGGUUATT-3’. siRNAs were transfected using Lipofectamine RNAiMAX Reagent according to the manufacturer’s protocol. After 48 h or 72 h transfection, cells were harvested and lysed to evaluate the transfection efficiency.

### Lentiviral infection

PHBLV-KAT7 and pHBLV-KAT7 mutant were constructed by cloning the full-length KAT7 and KAT7 mutant fragments into the XbaI /NotI sites of PHBLV-puro vector. Lentiviral constructs were transfected with packaging plasmids to HEK293T cells. The supernatant containing lentiviral particles was harvested at 48 h and filtered through a 0.45 μm filter, and then directly added to the culture medium. The infected cells were then selected with puromycin.

### Western blotting

Cells were washed twice with ice-cold 1×PBS, harvested, and lysed in radioimmune precipitation assay buffer (RIPA buffer; ApplygenTechnologies) with a phosphatase inhibitor tablet (Roche Diagnostics) and protease inhibitor mixture (Fermentas). Cell lysates were then centrifuged for 15 min at 15,000*g* at 4 °C, and the insoluble debris was discarded. Protein concentration was determined by using BCA protein assay reagent (Pierce). Cell lysates (20–40 μg) were subjected to 8–15% SDS-PAGE and transferred to nitrocellulose membranes (Millipore). The membrane was blocked using 5% milk in TBST buffer at room temperature for 1 h. Primary antibodies were blotted using 5% milk or BSA in TBST, and incubated at 4 °C overnight. The HRP-conjugated anti-mouse or anti-rabbit secondary antibodies were incubated for 1 h at room temperature in 5% milk/TBST. Then the signals were detected by enhanced chemiluminescence ECL (Pierce, Thermo Scientific), and imaged by films.

### Real-time PCR

Total RNA was extracted using the RNeasy Mini kit (Qiagen) following the manufacturer’s protocol and then subjected to reverse transcription using the StarScript first strand cDNA synthesis kit (Transgen Biotech, Beijing, China). Real-time PCR was performed using SYBR Select Master Mix (Applied Biosystems) on an ABI PRISM 7500 Sequence Detector (Applied Biosystems). GAPDH was served as an internal control for normalization. Results are representative of three independent experiments, and values are the mean ± SD (error bars). *P* < 0.05 (*) or *P* < 0.01 (**).

The primers for RT-qPCR are listed as below:

KAT7 forward: 5′-GAATGCAAGGTGAGAGCACA-3′;

KAT7 reverse: 5′-CCGTGTGTTCCCATAGGTCT-3′;

GAPDH forward: 5′-CCATGGGGAAGGTGAAGGTC-3′;

GAPDH reverse: 5′-GAAGGGGTCATTGATGGCAAC-3′.

### Immunoprecipitation

Cells were collected and lysed in IP lysis buffer (25 mM Tris-HCl (pH 7.4), 150 mM NaCl, 1% NP-40, 1 mM EDTA, and 5% glycerol) mixing with protease inhibitor cocktail (Sigma) at 4 °C for 30 min. The lysates were incubated with primary antibodies or control IgG overnight at 4 °C in rotation incubator followed by addition with protein G-Sepharose (GE Healthcare) at 4 °C for 2 h in rotation incubator. Samples were washed with IP lysis buffer for four times and PBS for one time. The immunoprecipitates were dissolved in 2×SDS loading buffer and subjected to 8–15% SDS-PAGE, then followed by western blotting.

### GST pull-down assay

GST and GST-tagged protein were expressed in BL21 (DE3) cells and affinity-purified with glutathione Sepharose 4B affinity chromatography according to the manufacture instructions. FLAG-PKD1-CA protein was expressed in HEK293T cells and purified with anti-FLAG affinity Beads (SMART) in accordance with the manufacture instructions. The purified FLAG-PKD1 (500 ng) and GST or GST-tagged protein (500 ng/each) were incubated together in 500 μL BC100 buffer at 4 °C overnight. Glutathione-sepharose beads (GE Healthcare) were added and incubated for 2–4 h at 4 °C. The beads were washed five times with BC100 buffer. The reaction mixture was boiled in Laemmli buffer. Western blotting was performed using antibody against FLAG and GST.

### In vitro kinase assay and identification of KAT7 phosphorylation sites by mass spectrometry

For in vitro kinase assay, 2 μg of GST-KAT7 and 8 μg of HA-PKD1-CA were incubated in kinase buffer (Cell Signaling Technology) for 30 min at 30 °C in the presence of 200 μM ATP. Then SDS loading buffer was added to stop the reaction. Phosphorylation of KAT7 was analyzed by Western blotting with anti-phosphoserine or anti-phosphothreonine antibodies.

To identify KAT7 phosphorylation sites, the reaction products were resolved by SDS-PAGE, and gels were stained with Coomassie Blue. The protein bands were retrieved and analyzed by mass spectrometry.

### Measuring protein half-life

HEK293T cells were transfected with plasmids as indicated. After 48 h transfection, 100 μg/ml cycloheximide (CHX) was added to the dishes, and the CHX treatment was terminated at 0, 2, 4, and 8 h time points as indicated. Whole cell lysates were prepared, and 25 μg of total protein from each sample was analyzed by Western blotting with anti-KAT7 antibody. Quantification of KAT7 protein was determined using Image J software and normalized to tubulin.

### In vivo ubiquitination

HEK293T cells were co-transfected with HA-tagged ubiquitin and other indicated plasmids for 42 h and cells were added with MG132 at final concentration of 20 μM for 6 h, then cells were collected and lysed. The samples were incubated with anti-Flag antibody in addition with protein G-Sepharose (GE Healthcare) and separated by SDS-PAGE and analyzed by western blotting.

### Cell growth curves

Cell proliferation was detected using 2-(2-Methoxy-4-nitrophenyl)-3-(4-nitrophenyl)-5-(2, 4-disulfophenyl)-2H-tetrazoliumsodiumsalt (CCK-8/WST-8) method. 1 × 10^3^ cells per well were seeded into 96-well plate and cultured for periods ranging from 1 to 7 day. The medium was changed every 24 h. At the indicated times, an aliquot of cells was stained with 10 μl of CCK-8 solution (Dojindo) for 1 h, and then the optical density at 450 nm was determined.

### Colony formation assay

To perform colony formation, 3 × 10^3^ and 1 × 10^4^ cells were cultured in six-well plate. After 10–14 days, cells were fixed in 4% (wt/vol) formaldehyde at 37 °C for 30 min and washed twice with 1×PBS, then stained with crystal violet for 1 h and washed twice with 1×PBS followed by photography.

### EdU incorporation assay

To perform Edu incorporation assay, 1 × 10^4^ cells were cultured in 96-well plate and performed assay with Cell-Light EdU Apollo567 In Vitro Kit (C10310-1, Ribobio) according to the manufacturer’s instructions. All cells were examined using fluorescence microscopy (Leica) with the appropriate filters. At least 300 cells were counted in randomly chosen fields from each culture well for statistical analysis.

### Statistical analysis

Two-tailed unpaired Student’s *t*-test was used to determine the significance of differences between samples indicated in figures. Results are depicted as mean values ± standard deviation (SD, *n* = 3). *P* < 0.05 (*) or *P* < 0.01 (**) were considered significant.

## Results

### PKD1 interacts with KAT7 in vitro and in vivo

**Fig. 1:**
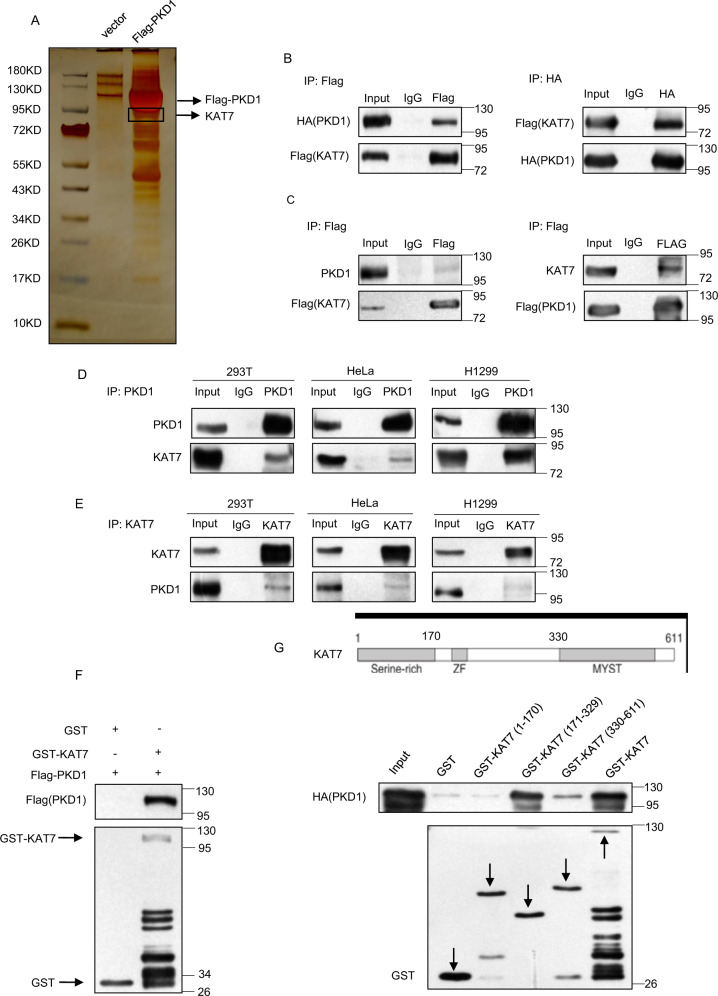
PKD1 interacts with KAT7 in vitro and in vivo. **a** KAT7 were pulled down by Flag-PKD1. Cellular extracts from HEK293T cells overexpressing FLAG-PKD1 were immunoprecipitated with anti-FLAG antibody and Protein G-Sepharose beads. After extensively washing the beads, immobilized proteins were eluted in SDS–PAGE sample buffer. The eluent was resolved by SDS–PAGE and silver stained. The protein bands were retrieved and analyzed by mass spectrometry. **b–e** PKD1 interacts with KAT7 in vivo. **b** HEK293T cells were co-transfected with HA-PKD1 and Flag-KAT7 plasmids. IP assay and subsequent western blotting were performed by using the indicated antibodies in the panel. **c** HEK293T cells were transfected with Flag-KAT7 and HeLa cells were transfected with Flag-PKD1, IP assay was carried out by using anti-FLAG antibody and followed by western blotting with anti-KAT7 or anti-PKD1 antibody, respectively. **d**, **e** Whole-cell lysates from HEK293T, HeLa, or H1299 cells were immunoprecipitated with anti-KAT7 or anti-PKD1 antibodies followed by immunoblotting with the indicated antibodies. **f** PKD1 interacts with KAT7 in vitro. GST pull-down assay were performed with bacterial expressed GST-KAT7 protein and HEK293T-expressed FLAG-PKD1. **g** The schematic representation of the key domains within KAT7 was depicted, and GST pull-down assay was performed by incubating GST-KAT7 or its various deletion mutants with HEK293T-expressed HA-PKD1.

In attempting to search for the interactors and potential novel substrates of PKD1, we ectopically expressed FLAG-PKD1 in HEK293T cells and immunoprecipitated FLAG-PKD1 by using Flag antibody. The eluted proteins were separated by SDS-PAGE and silver stained, and then subjected to LC–MS/MS analysis. Mass spectrometric analysis indicated that KAT7 was present in the PKD1 interactome (Fig. 1a).

To validate the interaction between PKD1 and KAT7, we co-transfected FLAG-KAT7 and HA-PKD1 into HEK293T cells and performed reciprocal co-immunoprecipitation (Co-IP) assays with anti-Flag or anti-HA antibodies followed by western blotting analysis. As shown in Fig. 1b, the exogenous KAT7 interacted with the exogenous PKD1 in HEK293T cells. We further observed that the endogenous PKD1 associated with the exogenously expressed FLAG-KAT7 in HEK293T cells and the endogenous KAT7 associated with the exogenously expressed FLAG-PKD1 in HeLa cells using Co-IP (Fig. 1c). Importantly, the interaction between the endogenous PKD1 and the endogenous KAT7 was also detected using reciprocal Co-IP in three cell lines including HEK293T, HeLa and H1299 cells, indicating that the two proteins interact with each other in vivo (Fig. 1d, e). Then the GST- pulldown assay was performed and confirmed that GST-KAT7, but not GST, pulled down FLAG-PKD1 in vitro (Fig. 1f), indicating that the interaction between PKD1 and KAT7 is direct.

To map the region of KAT7 responsible for binding to PKD1, we constructed a series of GST-tagged KAT7 deletion mutants either containing the N-terminal serine-rich domain (GST-KAT7 (1-170aa)), or the middle region containing a small zinc finger (ZF) domain (GST-KAT7 (171-329aa)), or the C-terminal region containing MYST domain with HAT activity (GST-KAT7 (330-611aa)), respectively (Fig. 1g, Supplementary Fig. 1). The GST-pulldown results showed that compared to the full-length KAT7, KAT7 (171-329aa) mutant had similar binding capacity to PKD1, KAT7 (330-611aa) mutant possessed weak binding to PKD1, whereas KAT7 (1-170aa) mutant had no binding ability to PKD1 at all (Fig. 1g). This result indicates that the 171-329aa region of KAT7 is critical for KAT7 binding to PKD1. Collectively, these results suggest that PKD1 directly interact with KAT7 in vitro and in vivo.

### KAT7 is phosphorylated at Thr97/Thr331 by PKD1 in vitro and in vivo

**Fig. 2:**
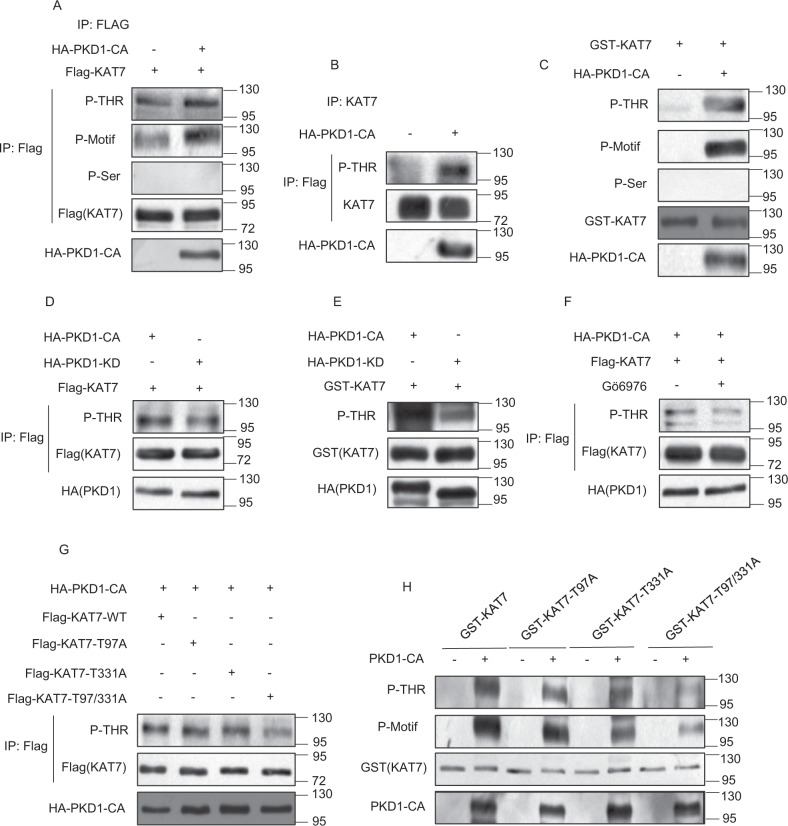
PKD1 phosphorylates KAT7 at Thr97/Thr331 in vitro and in vivo. **a** HEK293T cells were co-transfected Flag-KAT7 with or without HA-PKD1-CA. An IP assay was carried out using anti-Flag antibody followed by immunoblotting with anti-pMOTIF antibody, anti-phosphothreonine, anti-phosphoserine or anti-Flag antibodies, respectively. **b** HeLa cells were transfected with or without HA-PKD1-CA. An IP assay was carried out using anti-KAT7 antibody and immunoblotted with the indicted antibodies. **c** Bacterially expressed and purified GST (negative control) or GST-KAT7 was incubated with purified HA-PKD1-CA in a kinase reaction. The reaction products were separated by SDS-PAGE and immunoblotted with the anti-GST and the other indicated antibodies. **d** HEK293T cells were co-transfected Flag-KAT7 with HA-PKD1-CA or HA-PKD1-KD. An IP assay was performed and immunoblotted with the indicted antibodies. **e** Bacterially expressed and purified GST-KAT7 was incubated with purified HA-PKD1-CA or HA-PKD1-KD in a kinase reaction. The reaction products were separated by SDS-PAGE and immunoblotted with the indicated antibodies. **f** HEK293T cells were co-transfected with Flag-KAT7 and HA-PKD1-CA and treated with or without Gö6976. An IP assay and subsequent immunoblotting were performed as above. **g** HEK293T cells were cotransfected with WT-KAT7 or its mutants KAT7-T97A, T331A, or T97/331A and HA-PKD1-CA. An IP assay was performed and immunoblotted with the indicated antibodies. **h** Purified GST-tagged WT-KAT7 or its mutants were incubated with or without HA-PKD1-CA in a kinase reaction, and subsequent steps were performed as above.

Since PKD1 is a serine/threonine kinase, we next investigated whether PKD1 phosphorylates KAT7. To this end, FLAG-KAT7 was transfected with or without constitutive active PKD1 mutant HA-PKD1-CA (PKD1-S738/742E double mutations) in HEK293T cells. Then FLAG-KAT7 was immunoprecipitated by Flag antibody and subjected to immunoblotting analysis using PKD1 substrate motif antibody (p-Motif antibody) which recognizes PKD1-phosphorylated substrates at its consensus motif as well as antibodies recognizing phospho-serine residue or phospho-threonine residue (pan-p-Ser and pan-p-Thr antibodies), to detect KAT7 phosphorylation status in the PKD1-CA-overexpressing cells. The result showed that the exogenously expressed KAT7 could be phosphorylated by PKD1-CA in cells and KAT7 was threonine-phosphorylated but not serine-phosphorylated (Fig. 2a). The exogenously expressed FLAG-PKD1 was serine-phosphorylated by PMA treatment proved that the pan-p-Ser antibody was working (Supplementary Fig. 2). Furthermore, the endogenous KAT7 was also phosphorylated at threonine residue by PKD1-CA in HeLa cells (Fig. 2b). We also investigated KAT7 phosphorylation in vitro by incubation purified GST-tagged KAT7 with or without purified PKD1-CA in the presence of adenosine triphosphate (ATP). The result demonstrated that GST-KAT7 was efficiently phosphorylated at threonine residue by PKD1-CA in in vitro kinase assay, but not GST protein (Fig. 2c). In contrast, the kinase-inactive PKD1 mutant PKD1-KD (PKD1-K612W mutation) markedly mitigated KAT7 phosphorylation levels at threonine residue both in vivo and in vitro when compared with PKD1-CA (Fig. 2d, e). Similarly, PKD1 inhibitor Gö6976 treatment also attenuated the PKD1-CA-mediated KAT7 phosphorylation at threonine residue (Fig. 2f). Taken together, these results demonstrate that KAT7 is the substrate of PKD1 and PKD1 directly phosphorylates KAT7 at threonine residue.

Next, we performed in vitro phosphorylation of KAT7 and utilized mass spectrometry to identify which amino acid residue of KAT7 was phosphorylated by PKD1-CA. Two phosphorylated threonine sites including Thr97 and Thr331 were mapped by mass spectrometry in the presence of PKD1-CA. To validate whether PKD1 phosphorylates KAT7 at these sites, we mutated these two threonine residues to alanine individually or simultaneously to generate three mutants: KAT7-T97A, KAT7-T331A, and KAT7-T97/331A. The two single-residue mutants KAT7-T97A and T331A were still able to be threonine phosphorylated by PKD1-CA as similar levels as wide-type KAT7 (WT-KAT7) in vivo, whereas the double-residues mutant KAT7-T97/331A showed a significant reduction in threonine phosphorylation level when compared with wild-type KAT7 (Fig. 2g, Supplementary Fig. 3a), suggesting that PKD1-CA-mediated KAT7 threonine phosphorylation has been abolished by double mutation of T97/331 to A97/331. Moreover, the in vitro kinase assay results demonstrated that KAT7-T97A and T331A mutants preserved a significant partial of threonine phosphorylation by PKD1-CA relative to the WT-KAT7, while KAT7-T97/331A mutant almost completely lost the ability to be threonine phosphorylated by PKD1-CA (Fig. 2h, Supplementary Fig. 3b). Collectively, these results suggest that the Thr97 and Thr331 are two major sites in KAT7 phosphorylated by PKD1 in vivo.

### PKD1-mediated KAT7 phosphorylation promotes KAT7 protein expression

**Fig. 3:**
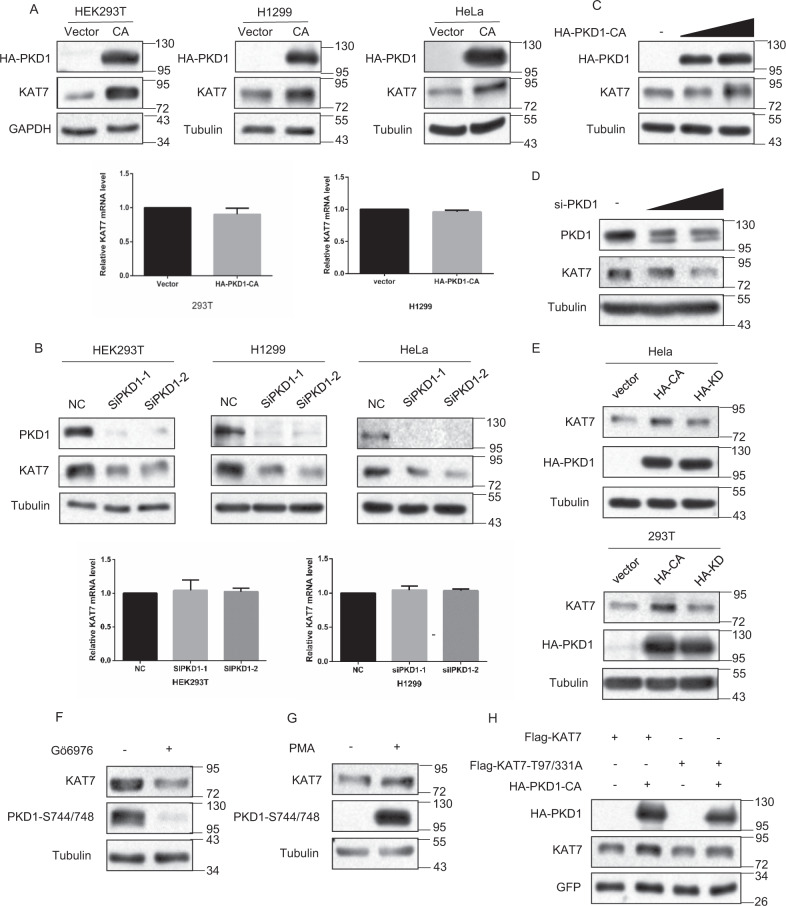
PKD1-mediated KAT7 phosphorylation increases KAT7 protein expression. **a** PKD1 overexpression increases KAT7 protein expression. HEK293T, H1299 and HeLa cells were transfected with either empty vector or HA-PKD1-CA. The total cell lysates were subjected to western blotting for the indicated proteins (upper panel). The total RNAs were extracted and the mRNA levels of KAT7 were measured by real-time PCR (lower panel). **b** PKD1 knockdown decreases KAT7 protein expression. The control siRNA or PKD1 siRNAs were transfected into HEK293T, H1299 and HeLa cells. The protein levels and mRNA levels of KAT7 were measured as above. **c**, **d** PKD1 regulates KAT7 expression in a dose-dependent manner. Different doses of HA-PKD1-CA or PKD1 siRNAs were transfected into HEK293T cells. The indicated proteins were measured by Western blotting. **e** PKD1 kinase activity is important for promoting KAT7 protein expression. The empty vector, HA-PKD1-CA or HA-PKD1-KD plasmids were transfected into HEK293T and HeLa cells, respectively. The indicated proteins were measured by Western blotting. **f** HEK293T cells were treated with or without Gö6976, then the indicated proteins were measured by Western blotting. **g** HEK293T cells were treated with or without PMA, then the indicated proteins were measured by Western blotting. **h** FLAG-KAT7 or KAT7-T97/331A mutant were transfected with or without HA-PKD1-CA into HEK293T cells, GFP plasmid also was transfected into all samples. The indicated proteins were measured by Western blotting. GFP protein levels are shown as equal transfection efficiency.

We then explored whether PKD1 affected KAT7 protein expression level. To this end, PKD1-CA was ectopically expressed in HEK293T, HeLa and H1299 cells, respectively. Compared to the vector control cells, PKD1-CA overexpression increased endogenous KAT7 protein expression levels in all three cell lines, but had no effect on KAT7 mRNA levels (Fig. 3a). In contrast, PKD1 knockdown by two independent siRNAs reduced KAT7 protein expression levels, and similarly, had no effect on KAT7 mRNA levels (Fig. 3b). Furthermore, we found that PKD1 modulated KAT7 protein expression in a dose-dependent manner (Fig. 3c, d).

We then analyzed whether the kinase activity of PKD1 played a role in regulating KAT7 protein expression. To test this, the empty vector, PKD1-CA or PKD1-KD plasmids were transfected into Hela and HEK293T cells, and then KAT7 protein levels were examined. Consistently, PKD1-CA overexpression markedly increased KAT7 protein expression levels compared to the vector control cells (Fig. 3e). However, PKD1-KD overexpression only slightly increased KAT7 protein levels (Fig. 3e). Similarly, PKD1 inhibition by Gö6976 treatment reduced KAT7 protein expression level, while PKD1 activation by PMA treatment elevated KAT7 level (Fig. 3f, g). These results indicate that the kinase activity of PKD1 is important for PKD1 to up-regulate KAT7 protein expression level.

As PKD1 increases KAT7 protein level dependent on its kinase activity, we next exploited whether PKD1-mediated KAT7 phosphorylation at Thr-97/Thr-331 is important for PKD1 to increase KAT7 protein level. PKD1-CA overexpression increased WT-KAT7 protein levels, but hardly promoted the unphosphorylatable mutant KAT7-Thr97/331A protein expression level (Fig. 3h). Collectively, these results suggest that PKD1-mediated KAT7 phosphorylation at Thr97/331 leads to the increase of KAT7 protein level, therefore, PKD1 regulates KAT7 protein expression at the posttranslational level.

It has been reported that PKD1 is activated by Nocodazole treatment^32^. Therefore, we asked whether KAT7 is phosphorylated by PKD1 in this process. Compared to the untreated cells, Nocodazole treatment remarkably stimulated PKD1 activation and induced ectopically expressed Flag-KAT7 phosphorylation at threonine residue in HEK293T cells (Supplementary Fig. 4a). In contrast, PKD1 knockdown by siRNA suppressed Nocodazole-induced threonine phosphorylation of Flag-KAT7, which suggests that PKD1 mediates Nocodazole-induced threonine phosphorylation of KAT7 (Supplementary Fig. 4b). Moreover, we also found that Nocodazole treatment increased KAT7 protein expression level (Supplementary Fig. 4c). Collectively, these results suggest that Nocodazole-stimulated PKD1 activation also phosphorylates KAT7 at threonine residue and regulates KAT7 protein expression.

### PKD1-mediated KAT7 phosphorylation enhances KAT7 protein stability by repressing KAT7 ubiquitination

**Fig. 4:**
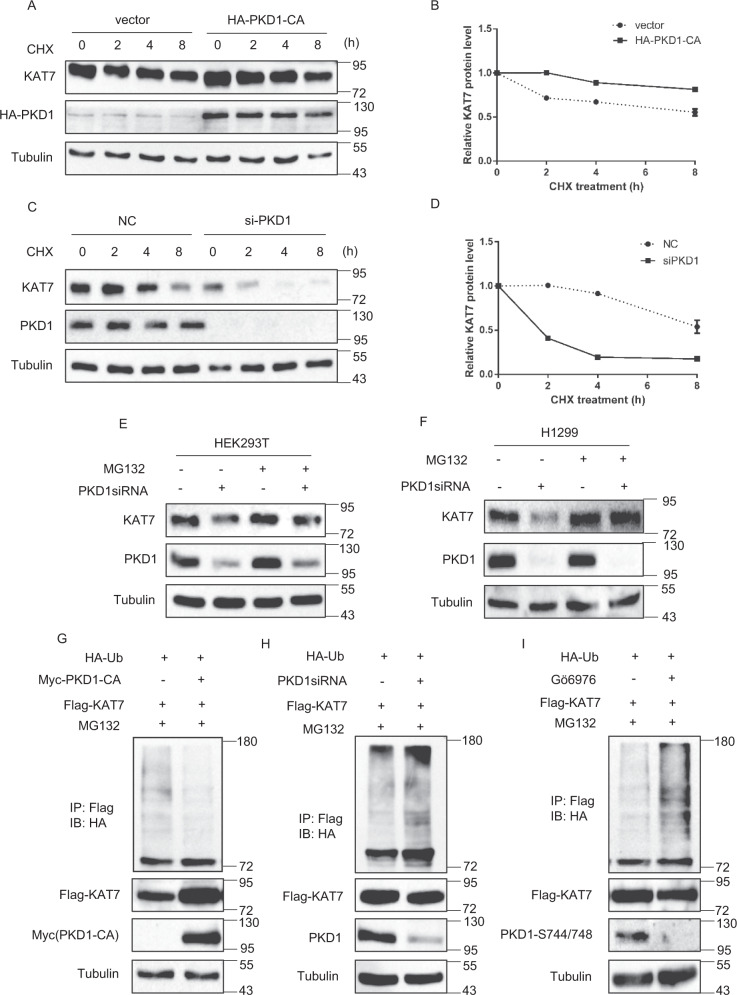
PKD1 prolongs the half-life of KAT7 protein and reduces KAT7 protein ubiquitination. **a** PKD1 overexpression increases the half-life of KAT7 protein. HEK293T cells were transfected with empty vector or HA-PKD1-CA plasmids for 36 h. Then 100 μg/ml cycloheximide (CHX) was added for the indicated time. The indicated proteins were detected by Western blot. **b** Data from **a** were quantified and graphed. **c** PKD1 knockdown decreases the half-life of KAT7 protein. HEK293T cells were transfected with PKD1 siRNA or control siRNA for 60 h. Then 100 ug/ml CHX was added for the indicated time. The indicated proteins were detected by Western blot. **d** Data from **c** were quantified and graphed. **e**, **f** PKD1 knockdown-induced KAT7 protein reduction is inhibited by proteasome inhibitor MG132. HEK293T and H1299 cells were transfected with PKD1 siRNA or control siRNA for 66 h, then cells were incubated with or without MG132 for another 6 h. The indicated proteins were detected by western blot. **g** PKD1 overexpression reduces KAT7 ubiquitination. HEK293T cells were co-transfected with Flag-KAT7, HA-Ub with or without Myc-PKD1-CA for 42 h and then treated with MG132 for another 6 h. Then IP assay was performed with Flag antibody and immunoblotted with HA antibody and other antibodies. **h** PKD1 knockdown enhances KAT7 ubiquitination. HEK293T cells were co-transfected with Flag-KAT7, HA-Ub, PKD1 siRNA or control siRNA for 42 h and then treated with MG132 for another 6 h. Then IP assay was performed with Flag antibody and immunoblotted with HA antibody and other antibodies. **i** 293 T cells were co-transfected with Flag-KAT7, HA-Ub for 42 h and treated with or without Gö6976, and then treated with MG132 for another 6 h. Then IP assay was performed with Flag antibody and immunoblotted with HA antibody and other antibodies.

We next investigated whether PKD1 promotes KAT7 protein expression by enhancing KAT7 protein stability. To test this, the empty vector and HA-PKD1-CA plasmids were transfected into 293T cells followed by cycloheximide (CHX) treatment to inhibit protein synthesis for the indicated time, and the endogenous KAT7 protein levels were then analyzed by immunoblotting. The results demonstrated that the half-life of endogenous KAT7 in the empty vector-expressed cells was over 8 h (Fig. 4a, b). PKD1-CA overexpression significantly increased the half-life of KAT7 (Fig. 4a, b). By contrast, PKD1 knockdown by siRNA drastically reduced the half-life of KAT7 to less than 2 h (Fig. 4c, d). These results imply that PKD1 promotes KAT7 protein levels by enhancing its protein stability.

To determine whether PKD1 knockdown-induced KAT7 protein down-regulation is proteasome dependent, the siRNA-PKD1-transfected HEK293T and H1299 cells were exposed to the proteasome inhibitor MG132. MG132 treatment substantially reversed PKD1 knockdown-induced down-regulation of KAT7 (Fig. 4e, f). These results indicate that PKD1 deficiency-induced KAT7 protein degradation is mediated by the ubiquitin-proteasome pathway.

We next examined whether PKD1 affects the ubiquitination of KAT7. To this end, Flag-KAT7, HA-tagged ubiquitin with or without Myc-PKD1-CA plasmids were co-transfected into HEK293T cells and then treated with MG132. The cell lysates were immunoprecipitated with Flag antibody and immunoblotted with HA antibody. Significant amount of slowly migrating smeared bands behind Flag-KAT7 signal were detected in the absence of PKD1-CA, suggesting that Flag-KAT7 was ubiquitinated (Fig. 4g). PKD1-CA overexpression significantly decreased KAT7 ubiquitination (Fig. 4g). Conversely, PKD1 knockdown markedly increased the ubiquitination of KAT7 compared to the control cells (Fig. 4h). Similarly, PKD1 inhibition by Gö6976 treatment also largely increased KAT7 ubiquitination (Fig. 4i). Taken together, these results suggest that PKD1 prolongs the half-life of KAT7 protein by inhibiting its ubiquitination-mediated degradation.

### PKD1-mediated threonine phosphorylation of KAT7 promotes pre-RC formation and cell proliferation

**Fig. 5:**
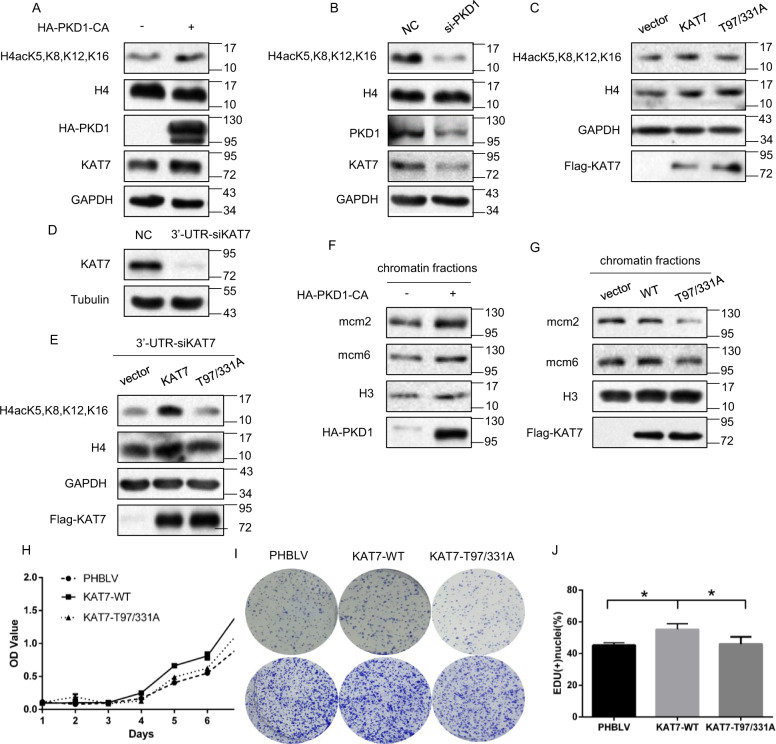
PKD1-mediated phosphorylation of KAT7 at T97/331 promotes pre-RC formation and cell proliferation. **a** Phosphorylation of KAT7 by PKD1 is important for pre-RC formation. HeLa cells transfected with or without HA-PKD1-CA were subjected to western blot for the indicated proteins. **b** HeLa cells transfected with PKD1 siRNA or control siRNA were subjected to western blot for the indicated proteins. **c** HeLa cells transfected with vector, KAT7-WT and KAT7-T97/331A mutant were subjected to western blot for the indicated proteins. **d** HeLa cells transfected with KAT7 3’-UTR siRNA or control siRNA were immunoblotted with KAT7 antibody to examine KAT7 knockdown efficiency. **e** HeLa cells were transfected with KAT7 3’-UTR siRNA for 24 h, then re-introduced with vector, WT-KAT7 and KAT7-T97/331A mutant for another 48 h. Cell lysates were subjected to western blot for the indicated proteins. **f** HeLa cells transfected with or without HA-PKD1-CA were lysed and separated to cytoplasmic, soluble nucleoplasmic and chromatin-enriched fractions, respectively. Then chromatin-enriched fractions were subjected to western blot analysis for the indicated proteins. **g** HeLa cells transfected with vector, WT-KAT7 and KAT7-T97/331A mutant were lysed and separated to cytoplasmic, soluble nucleoplasmic and chromatin-enriched fractions, respectively. Then chromatin-enriched fractions were subjected to western blot analysis for the indicated proteins. **h** HeLa cells were stably infected with PHBLV, KAT7-WT and KAT7-T97/331 A lentiviruses, respectively, and then cell growth curves were determined by CCK-8/WST-8 assay for the indicated time. **i** HeLa cells infected as **h** were cultured for 10–14 days, then colony formation assay was performed. **j** Hela cells were infected as **h**, and then EdU incorporation assay was conducted.

As KAT7 is a HAT enzyme responsible for histone H4 acetylation in vivo^20,22^, we sought to exploit whether PKD1-mediated threonine phosphorylation of KAT7 affects histone H4 acetylation. To this end, we utilized the antibody which could detect acetylation modifications at four lysine residues of histone H4, including K5, 8, 12 and 16. As shown in Fig. 5a, overexpression of PKD1-CA increased histone H4 acetylation levels at K5, 8, 12 and 16 compared to the vector control cells. Conversely, silencing of PKD1 by siRNA reduced histone H4 acetylation levels (Fig. 5b). Moreover, phospho-defective mutant KAT7-Thr97/331A overexpression also slightly lowered histone H4 acetylation levels when compared with WT-KAT7-expressing cells (Fig. 5c). To eliminate the endogenous KAT7 effect and further validate this result, we utilized KAT7 3′-UTR siRNA (si-KAT7 UTR) that targeting to KAT7 mRNA 3′-UTR to knockdown KAT7 first (Fig. 5d), and then re-introduced empty vector, WT-KAT7 or KAT7-T97/331A mutant into cells, respectively. Compared to the vector control cells, re-expression of WT-KAT7 remarkably enhanced histone H4 acetylation levels, whereas KAT7-T97/331A mutant was unable to increase histone H4 acetylation level (Fig. 5e). Collectively, these results indicate that PKD1 increases histone H4 acetylation levels through phosphorylation of KAT7 at Thr97/331.

KAT7 promotes loading of the MCM2-7 complex on the chromatin by acetylating histone H4^18–22^. We then examined the effects of PKD1-mediated threonine phosphorylation of KAT7 on the chromatin loading of MCM proteins. To this end, HA-PKD1-CA, or Flag-KAT7, or Flag-KAT7-T97/331A constructs were transfected into cells, and then chromatin-bound fraction was separated and subjected to western blot analysis. Compared to the vector control cells, PKD1-CA overexpression largely increased the loading of MCM2/MCM6 on the chromatin (Fig. 5f). Moreover, phospho-defective mutant KAT7-Thr97/331A significantly reduced the abundances of chromatin-bound MCM2/MCM6 when compared with WT-KAT7-expressing cells (Fig. 5g). Altogether, these results suggest that PKD1 facilitates the loading of MCM proteins on the chromatin via phosphorylation of KAT7 at Thr97/331.

As KAT7 is essential for DNA replication and regulates cell proliferation^18–20,26^, we further analyzed whether PKD1-mediated threonine phosphorylation of KAT7 affects cell proliferation. Compared to the vector control cells, WT-KAT7 overexpression was more potent in promoting cell proliferation as measured by CCK-8 assay and colony formation assay, whereas KAT7-T97/331A mutant substantially lost the ability to promote cell proliferation (Fig. 5h, i). Furthermore, KAT7-T97/331A overexpression reduced EdU incorporation ratio as measured by EdU incorporation assay, namely decreased the DNA synthesis, when compared with WT-KAT7 (Fig. 5j). Collectively, these results indicate that PKD1-mediated phosphorylation of KAT7 at Thr97/331 enhances DNA replication and cell proliferation.

## Discussion

Several lines of evidence have suggested that KAT7 is subjected to the phosphorylation modification in response to the environment or cell signaling cues. During mitosis, CDK1 and PLK1 sequentially phosphorylate KAT7 on Thr85/88 and Ser57, respectively, which is required for pre-RC formation and DNA replication licensing^27^. ATM/ATR-dependent phosphorylation of KAT7 at Ser50/53 facilitates nucleotide excision repair and cell survival under UV irradiation^28,29^. The cyclin E/CDK2 complex phosphorylates KAT7 at Thr88 which promotes the enrichment of breast cancer stem-like cells^30^. In this study, we have found that PKD1 directly interacts with KAT7 and phosphorylates KAT7 at two threonine residues T97/331 in vitro and in vivo. We further reveal that the 171-329aa region of KAT7 containing a small zinc finger (ZF) domain is critical for KAT7 binding to PKD1. Thus, we have identified that KAT7 is a novel substrate of PKD1 and PKD1 is a novel upstream kinase to phosphorylate KAT7.

Protein phosphorylation is a key posttranslational modification that regulates protein activity, stability, and localization^33^. Several reports have suggested that KAT7 phosphorylation plays an important role in KAT7 protein stability via affecting its ubiquitination^28,31^. LPS exposure-induced activation of MEK1 phosphorylates KAT7, thereby facilitating ubiquitin E3 ligase FBXW15 recruitment to promote KAT7 degradation^31^. UV irradiation-triggered KAT7 phosphorylation by ATM/ATR is preferentially ubiquitinated by ubiquitin E3 ligases CRL4-DDB2 to promote KAT7 degradation^28^. In contrast to these studies that KAT phosphorylation facilitates its ubiquitination and subsequent degradation, our results indicate that phosphorylation of KAT7 at Thr97/331 by PKD1 increases KAT7 protein expression levels by enhancing KAT7 protein stability via inhibiting its ubiquitination and subsequent degradation. We speculate that PKD1-mediated KAT7 phosphorylation may affect the function of ubiquitin E3 ligase or may interfere with the interaction between KAT7 and E3 ligase. However, the underlying molecular mechanism how PKD1 regulates KAT7 ubiquitination needs to be further elucidated.

KAT7 is the major enzyme responsible for acetylation of histone H4 and H3 in vivo to promote the loading of MCM complex on the chromatin and initiates DNA replication^18–24^. KAT7 also positively regulates cell proliferation under normal cell growth conditions through above epigenetic mechanism. Previous studies have defined a role for PKD1 in DNA synthesis and cell proliferation via an increased duration of the ERK signaling and entry into the S phase of the cell cycle^6,34,35^. Here we found that PKD1-CA could directly enhance histone H4 acetylation levels and MCM2/6 loading on the chromatin, whereas PKD1 knockdown largely mitigate histone H4 acetylation levels. Moreover, compared to the WT-KAT7, PKD1 unphosphorylatable mutant KAT7-Thr97/331A substantially lose the ability to acetylate histone H4 and promote the loading of MCM2/6 on the chromatin. Consequently, KAT7-Thr97/331A mutant significantly reduce the cell proliferation and DNA synthesis relative to the WT-KAT7. Therefore, we define a novel mechanism that PKD1 promotes cell proliferation and DNA synthesis, namely PKD1-mediated phosphorylation of KAT7 at Thr97/331 to promote pre-RC formation, DNA replication and cell proliferation.

Regulation of chromatin accessibility via acetylation/deacetylation of nucleosome histones is an important epigenetic mechanism controlling gene expression. Class-IIa histone deacetylases (HDAC4, HDAC5, HDAC7 and HDAC9) regulate chromatin structure by interacting with various transcription factors, such as myocyte enhancer factor 2 (MEF2), RUNX and CAMTA2 to repress their target genes transcription^36^. Mounting evidences show that activated PKD1 directly phosphorylates HDAC5 and HDAC7 in the nucleus, leading to their dissociation from the transcription factors and nuclear export, thereby de-repressing the target genes transcription to regulate diverse biological process^2,37–41^. However, whether PKD1 can phosphorylate histone acetyltransferases (HATs) to regulate cell functions remains unknown. Here we found that PKD1 directly phosphorylates a HAT enzyme KAT7, leading to the decrease of its ubiquitination and the increase of its protein stability and expression level, thereby enhancing histone H4 acetylation levels and MCM2/6 loading on the chromatin, ultimately promoting DNA replication and cell proliferation. To our knowledge, this is the first time to unveil that PKD1 can directly phosphorylates HAT enzyme to modulate biological functions at epigenetic level. However, future studies are needed to further analyze the impact of PKD1-mediated phosphorylation of KAT7 on the HAT activity of KAT7, KAT7 localization, and the integrity of KAT7 complexes.

In conclusion, we demonstrate that KAT7 is a substrate of PKD1 and PKD1-mediated phosphorylation of KAT7 at Thr97/331 decreases its ubiquitination, which in turn enhances KAT7 protein stability and expression, thereby promoting histone H4 acetylation, pre-RC formation, DNA replication and cell proliferation.

## Supplementary information

Supplemental Information

Supplementary Fig 1

Supplementary Fig 2

Supplementary Fig 3

Supplementary Fig 4
