# Supplementary material for: Protein kinase D1 phosphorylation of KAT7 enhances its protein stability and promotes replication licensing and cell proliferation
Source: Cell Death Discov. 2020 Sep 18;6:89. doi: 10.1038/s41420-020-00323-w (PMC7501302; doi:10.1038/s41420-020-00323-w)
Supplement: Supplementary file 1 — Supplemental Information [file 41420_2020_323_MOESM1_ESM.docx]

**Supporting Information**

**Protein kinase D1 phosphorylation of KAT7 enhances its protein stability and promotes replication licensing and cell proliferation**

Yao Liang^1^, Yuanyuan Su^1^, Chenzhong Xu^1^, Na Zhang^1^, Doudou Liu^1^, Guodong Li^1^, Tanjun Tong^1^, Jun Chen^1,^*

^1^Peking University Research Center on Aging, Beijing Key Laboratory of Protein Posttranslational Modifications and Cell Function, Department of Biochemistry and Molecular Biology, Department of Integration of Chinese and Western Medicine, School of Basic Medical Science, Peking University, Beijing 100191, China.

*Correspondence

Jun Chen

Tel: 86-10-82802527; Fax: 86-10-82802931; E-mail: [cjbiochem@bjmu.edu.cn](mailto:cjbiochem@bjmu.edu.cn).

**Supplementary Figure Lengends**

**Supplementary Fig. 1 The expressions of GST-KAT7 and its deletion mutants (related to Fig. 1g).**

(**a**) The purified recombinant GST-tagged full-length KAT7 and its various deletion mutants were separated on SDS-PAGE gel and stained with coomassie blue.

**Supplementary Fig. 2 PKD1 serine-phosphorylation was detected by pan-p-Ser antibody (related to Fig. 2a).**

(**a**) HEK293T cells were transfected with Flag-KAT7 or empty vector. After 48 h transfection, the Flag-KAT7-expressed cells were treated with 10 nM PMA for 10 min. Then the cells were collected and an IP assay was carried out using anti-Flag antibody followed by immunoblotting with anti-pan-p-Ser or anti-Flag antibodies, respectively.

**Supplementary Fig. 3 PKD1 phosphorylates KAT7 at Thr97/331 (related to Fig. 2g, h).**

(**a**) HEK293T cells were co-transfected with WT-KAT7 or its mutants KAT7- T97/331A and with or without HA-PKD1-CA. An IP assay was performed and immunoblotted with the indicated antibodies. (**b**) Purified GST-tagged WT-KAT7 or its mutant KAT7- T97/331A were incubated with or without HA-PKD1-CA in a kinase reaction. The reaction products were separated by SDS-PAGE and immunoblotted with the indicated antibodies.

**Supplementary Fig. 4 Nocodazole-stimulated PKD1 activation phosphorylates KAT7 at threonine residue and regulates KAT7 protein expression (related to Fig. 3).**

(**a**) HEK293T cells were transfected with Flag-KAT7 and treated with or without Nocodazole (100 ng/mL). An IP assay was carried out using anti-Flag antibody followed by western blotting with anti-phosphothreonine or anti-Flag antibodies. (**b**) HEK293T cells were co-transfected with Flag-KAT7 and PKD1 siRNA or control siRNA, and then treated with or without Nocodazole. An IP assay was carried out using anti-Flag antibody followed by western blotting with anti-phosphothreonine or anti-Flag antibodies. (**c**) HEK293T cells treated with or without Nocodazole, then the protein levels of KAT7 were analyzed by western blotting.
