# Supplementary figures and images for: Protein kinase D1 phosphorylation of KAT7 enhances its protein stability and promotes replication licensing and cell proliferation
Source: Cell Death Discov. 2020 Sep 18;6:89. doi: 10.1038/s41420-020-00323-w (PMC7501302; doi:10.1038/s41420-020-00323-w)

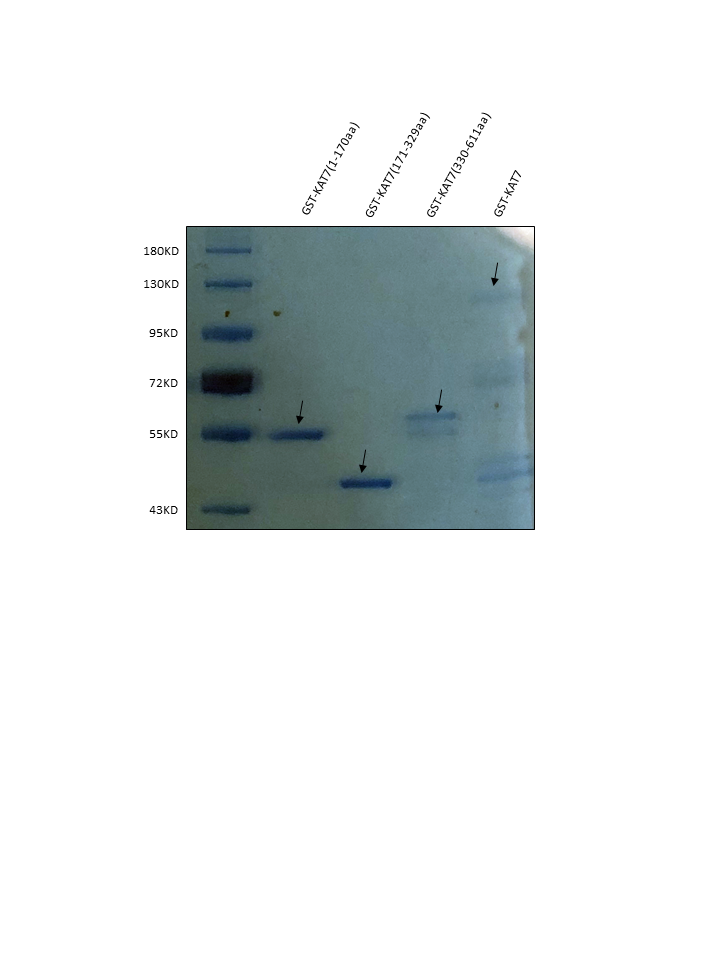

Supplement: Supplementary file 2 — Supplementary Fig 1 [file 41420_2020_323_MOESM2_ESM.tif]

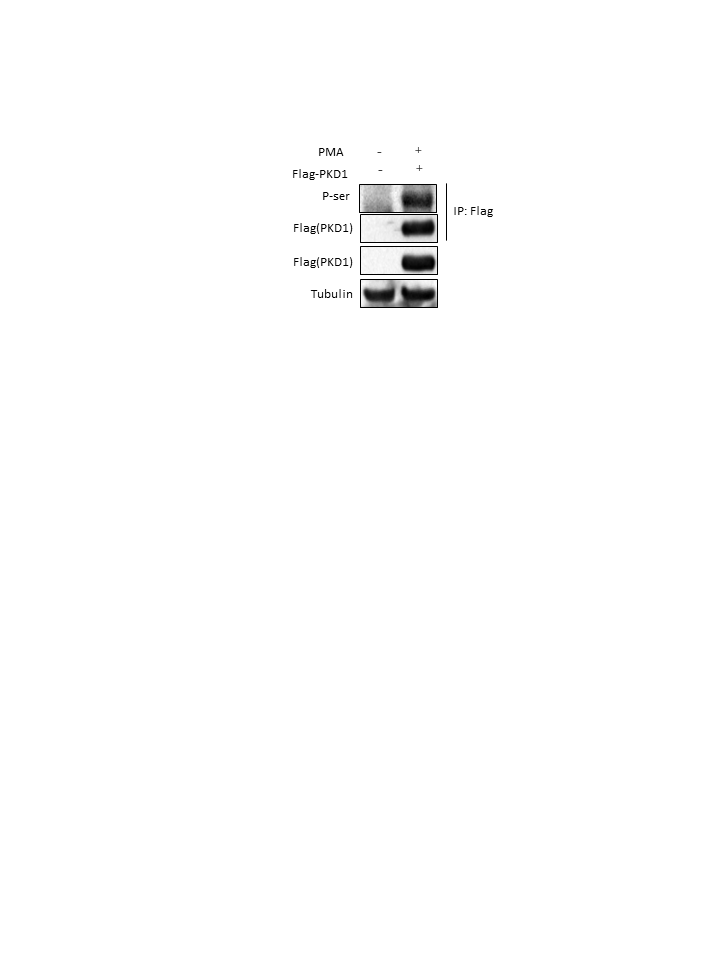

Supplement: Supplementary file 3 — Supplementary Fig 2 [file 41420_2020_323_MOESM3_ESM.tif]

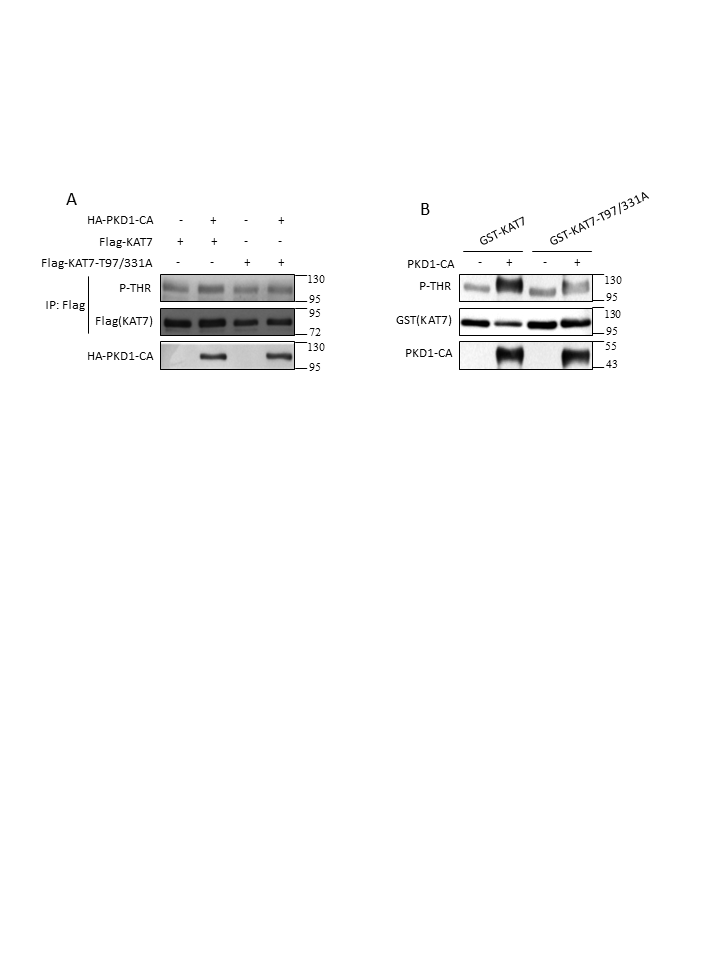

Supplement: Supplementary file 4 — Supplementary Fig 3 [file 41420_2020_323_MOESM4_ESM.tif]

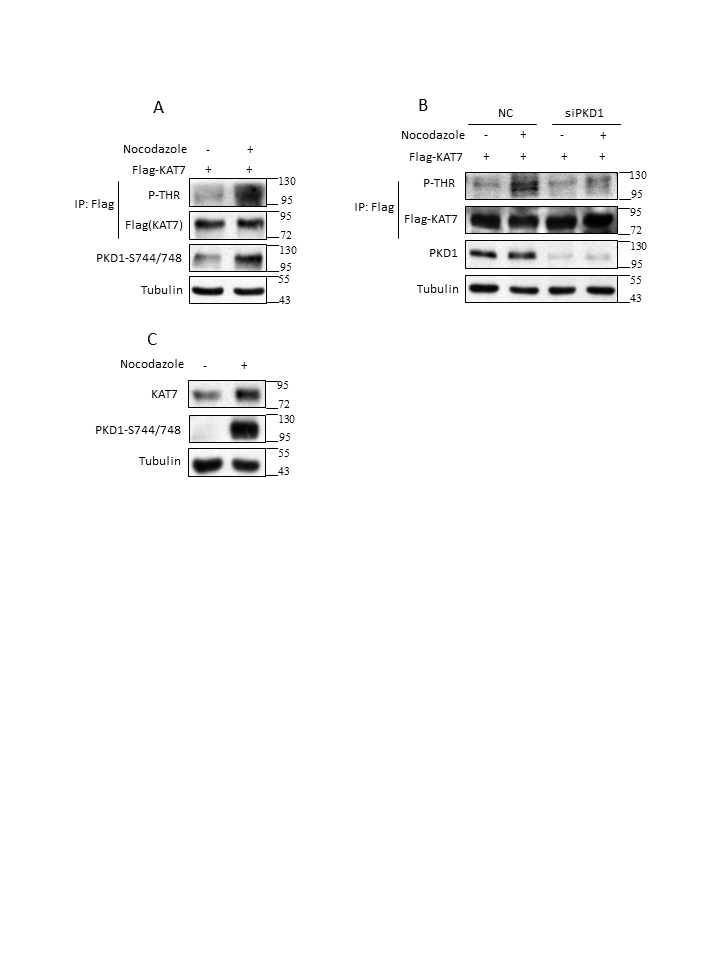

Supplement: Supplementary file 5 — Supplementary Fig 4 [file 41420_2020_323_MOESM5_ESM.tif]
